# Effect of Light Regiment on Farrowing Performance and Behavior in Sows

**DOI:** 10.3390/ani11102858

**Published:** 2021-09-30

**Authors:** Shelby McLoda, Nichole C. Anderson, Jennifer Earing, Drew Lugar

**Affiliations:** 1 Department of Agriculture, Illinois State University, Normal, IL 61761, USA; samclod@ilstu.edu (S.M.); jeearin@ilstu.edu (J.E.); 2 School of Veterinary Medicine, Texas Tech University, 7671 Evans Drive, Amarillo, TX 79106, USA; Nichole.Anderson@ttu.edu

**Keywords:** farrowing, light cycle, photoperiod, sow

## Abstract

**Simple Summary:**

When sows approach birthing in most commercial farms, they are often exposed to continuous light to help farm workers monitor pig health and progress. Being in constant lighting may have a negative effect on sows as mammals often prefer a quiet environment with low stimulation during birthing. In our study, we placed 15 sows in a 12 h light/12 h dark environment, and 15 sows in 24 h light. Sows were watched for ease of farrowing (measured in total time farrowing, birthing interval, and behavior during farrowing). We also measured total piglets born alive and stillborn piglets. Overall, we observed that sows who received darkness gave birth earlier in gestation. Light availability had no effect on total piglets born alive, farrowing behavior, or total length of time giving birth. It was observed that there were more stillborn animals for the sows who received darkness. Overall, it is possible that light availability could have an impact on farrowing behavior and health; however, more factors should be considered in the environment.

**Abstract:**

The purpose of this study was to investigate the effects of light exposure on farrowing performance in sows. Thirty sows were moved to the farrowing unit at d 110 of gestation and assigned a treatment: 12 h light/12 h dark cycle (Dark) or 24 h light (Light). Treatments began upon entry into the farrowing unit. Video was recorded continuously from initiation of the treatments until completion of farrowing. Data collected included duration of farrowing, birthing interval, and behavior during farrowing. Additionally, the number of total born, liveborn, and stillborn piglets was recorded. Gestation length was different between treatments, with a shorter gestation in Dark treatment sows than Light treatment sows (116.4 vs. 117.1 ± 0.2 d, respectively; *p* = 0.027). The total duration of parturition and number of liveborn did not differ (*p* = 0.393). Number of stillborn piglets between treatments did differ (*p* = 0.018). Dark had more stillborns compared to Light treatment sows (1.5 vs. 0.7 ± 0.2 piglets, respectively). Neither the interval between piglets nor farrowing behavior differed between treatments (*p* > 0.100). The results from this experiment indicate that a sudden change in photoperiod has the potential to impact the gestation length of sows and number of stillborn pigs.

## 1. Introduction

Sows on commercial swine operations are exposed to varying amounts of light in the form of heat lamps and/or overhead lighting. The duration of light exposure for farrowing sows varies by operation, with many sows being exposed to overhead light for 8–10 h per day and heat lamp light for 24 h per day. The light cycle an animal is exposed to has been shown to have an impact on the preferred time of parturition in horses [1], rats [2], and alpaca [3].

Though there has been much research on the effects of photoperiod on mammals, little if any research has investigated the effects of photoperiod on the duration of parturition and the associated complications of parturition. However, it has been suggested that pigs have low regularity when it comes to their response to photoperiod [4]. Many of the farrowing complications observed on commercial sow farms can be attributed to farrowing duration [5]. In a study by Mainau et al. [5], correlations between parturition duration and the incidence of stillborn piglets and increased piglet mortality were observed. Therefore, the purpose of this study was to evaluate the duration of farrowing, birthing interval, number of stillborn piglets of sows exposed to either continuous light or a 12 h light cycle during farrowing. The hypothesis of the present study was that sows exposed to a 12 h light cycle would have a reduced farrowing duration and fewer stillborn piglets.

## 2. Materials and Methods

All experimental procedures were approved by the Institutional Animal Care and Use Committee at Illinois State University (#2019-1131).

### 2.1. Experimental Design and Animals

This study was conducted at the Illinois State University Farm and utilized 30 pregnant, crossbred (Yorkshire × Chester White) sows. Sows were housed in conventional farrowing stalls and randomly allocated to one of two light regiment treatments at d 110 of gestation. Light regiments included 12 h of light/12 h dark per day (Dark) or 24 h of light per day (Light). Previous work has demonstrated that a 12 h dark period increases melatonin levels in swine, which provides an indicator of sufficient light to dark differences in the pig’s experience [6]. Sows were equally allotted to light treatment based on sow parity (2.14 ± 0.53 parities and 2.00 ± 0.65 parities for Dark and Light, respectively). Sow parity ranged from 1 to 3 prior to farrowing, where all sows had previously delivered at least 1 L prior to the present study. The study was conducted in two replicates with an equal number of sows per treatment in each replicate. Sows of both treatments were housed in the same farrowing room for both replicates. Prior to initiation of the study, sows were exposed to 24 h of light per day from breeding until initiation of lighting treatments at 110 d of gestation. Due to the condition sows were housed in prior to this study, the Light treatment represents the control group. Overhead lights were left on for 24 h per day and manipulation of light for sows in the Dark treatment was achieved by use of light deprivation tarps (9 mil, Tunnel Vission Hoops LLC, Shaker Heights, OH, USA) that were suspended from the ceiling to the top of the farrowing crate dividers. To ensure proper ventilation, a commercial 10.16 cm drainage tile was placed between the farrowing crate dividers and the light deprivation tarps. Staggering 2.54 cm diameter holes were drilled on either side of the drainage tile for ventilation of the farrowing crate in the Dark sows. For Dark sows, light deprivation tarps were lowered from the ceiling at 19:00 h daily from d110 of gestation until completion of farrowing. Each day at 07:00 h, the light deprivation tarps were lifted. In order to minimize the amount of light in the Dark farrowing crates, heat mats were utilized for supplemental heat for both treatment groups. When the light deprivation tarps were up (light phase), they were rolled toward the ceiling. The rolled tarps extended approximately 30.5 cm from the ceiling. Prior to initiation of the present study, illuminance was measured in a pilot study to determine the effectiveness of light deprivation from the tarps. For this, a digital illuminance meter (AOPUTTRIVER AP-881E) was utilized to measure the amount of light in the center of the sow stall approximately 30.5 cm from the front end of the crate at three heights 30.5, 61.0, and 91.4 cm from the flooring. Illuminance data are summarized in Table 1.

Video cameras (LBV2531 cameras with D841A63B DVR, Lorex Technology, Markham, ON, CA) were installed above each farrowing crate and continuously recorded video from day 110 of gestation until completion of farrowing, which was defined as the time at which the first piglet emerged from the sow. Behavior observations were performed as scan samples from video every 5 min by a single, trained observer. Observations began two hours prior to farrowing (‘prior to farrowing’; start of farrowing was defined as when the first piglet emerged from the sow) and continued until the completion of farrowing (‘during farrowing’; completion of farrowing defined as when the last piglet emerged from the sow). Behaviors were adapted from Chapel et al. [7] and Van Beirendonck et al. [8] and can be reviewed in an ethogram in Table 2. Additionally, video footage was used to measure duration of farrowing, birthing interval, and gestation length, total born and liveborn piglets were recorded for each sow. All sows received the same light treatment following the completion of parturition.

### 2.2. Statistical Analysis

Two repetitions of this study were conducted with a total of 15 sows per treatment represented. However, due to a technical issue, data is missing from one sow in the Dark treatment, thus statistical analysis was performed on 14 Dark treatment sows and 15 Light treatment sows. All variables were analyzed using the Mixed procedure of SAS (v9.4; SAS Inst. Inc., Cary, NC, USA). The main effect in the model included light treatment. Replicate of the study was utilized as a random effect, and average litter size prior to the trial was used as a covariate, when appropriate. Duration of farrowing was standardized for each sow in order to account for the number of liveborn and stillborn piglets. This was accomplished by taking the total duration of farrowing, divided by the sum of the liveborn and stillborn piglets from that sow and multiplying that by 12.2 (average number of liveborn and stillborn piglets in the present study). Mummified piglets were not included in this standardization due to the fact that they could not be observed in the video recordings and pass easily through the reproductive tracts of the sows. For interval between piglet births, a repeated measures analysis was utilized with a compound symmetry covariant structure. Statistical significance was defined as *p* ≤ 0.05.

## 3. Results

Farrowing performance results are summarized in Table 3. Dark treatment sows had a shorter gestation length (116.4 ± 0.8 d) compared to Light treatment sows (117.2 ± 0.8 d; *p* = 0.026). Duration of parturition (adjusted for live and stillborn litter size) and the interval between piglets did not differ between treatments (*p* ≥ 0.393). Number of liveborn piglets, mummified piglets and total born did not differ between treatments (*p* ≥ 0.459), however the number of stillborn piglets between treatments were different (*p* = 0.018). Dark treatment sows had an increased incidence of stillborn piglets (1.5 ± 0.2 piglets/L) compared to Light treatment sows (0.7 ± 0.2 piglets/L). No differences were observed for behavior including postures and posture change, both prior to and during farrowing (*p* ≥ 0.101; Summarized in Table 4).

## 4. Discussion

The sows on the present study had been accustomed to continuous overhead lighting, which is the current practice at the Illinois State University farm in the breeding, gestation, and farrowing units. Melatonin, ACTH, and cortisol rhythms are constant when animals are housed in continuous light conditions creating a “free-running circadian rhythm” [9]. It is possible that the sows were in a free-running circadian rhythm prior to the trial and throughout the trial for the Light group. The shift from continuous light to a 12 h light cycle may have altered the secretion pattern of melatonin in the Dark sows. Melatonin, in particular, plays a significant role on reproduction in the pig and acts as a major cause of seasonal infertility in swine [6]. McConnell and Ellendorff [10] reported that sows on a 12 h light cycle had a scotophase surge of melatonin, but sows on a long or short duration light cycle did not. It has been suggested that sows do not respond normally compared to other species to sudden changes in their light cycle [6], which could have altered the Dark sows’ melatonin, and (or) cortisol secretion patterns in the present study.

In the present study, the shift from the practice of continuous light in the Dark sows resulted in a shorter gestation length. The average gestation length at the Illinois State University farm is 117.2 days from the time of the first observed estrus and insemination, whereas Dark sows gave birth approximately one day before expected based on the herd average. While there has been limited research on the effects of light cycles on the gestation length in sows, there have been studies in other species, with variable results. Nolan et al. [11] reported that artificially extending the daylength in mares late in gestation results in a reduction in gestation length. However, Malschitzky et al. [12] reported no effect of photoperiod on gestation length in mares. The differences in these equine studies may be a result of the type of light utilized; Malschitzky et al. [12] utilized incandescent bulbs while Nolan et al. [11] utilized short wavelength blue light to a single eye. Similar work has shown no impact in alpaca gestational length [3]. Sudden variations in light: dark cycles appears to be stressful for mammals [13]. A study in mice that investigated repeated shifts in light cycles reported that the shifting of light cycles is stressful to the animals [14]. Additionally, a sheep model was utilized to investigate the effect of shift work on pregnancy and resulted in an extended gestation length [15]. In this study, the light cycle of was alternated twice weekly for the treatment ewes whereas the control ewes were exposed to a constant 12 h light cycle. In the present study, altering the light cycle from continuous light to a 12 h light cycle, resulted in a shortened gestation length. To the authors’ knowledge, this is the first evidence that altering the light regiment for sows may impact their gestation length.

The hypothesis of the present study was that Dark sows would have a reduced duration of farrowing and reduced interval between piglet births, based on the prediction that darkness would reduce stress experienced by the sow, allowing sows to complete farrowing rapidly. However, the present study reported that the total duration of parturition and the interval between piglets did not differ between treatments. This result may be explained by the relatively small number of sows utilized and the high amount of variation in duration of parturition. While the number of liveborn piglets between treatments did not differ in the present study, the number of stillborn piglets were greater in Dark sows compared to Light sows in the present study. A study conducted by Oliviero et al. [16] found that environmental factors such as the type of farrowing enclosure affects the duration of parturition and interval between piglets. Though light manipulation did not occur in that study, the authors reported that the duration of farrowing was longer and there was a longer interval between piglet births for sows that gave birth in crates compared to pens. It was suggested that the stress of confinement resulted in an increased release of opioids, which resulted in a reduced oxytocin concentration. Stress events result in the release of corticotropin releasing hormone which in turn causes the release of ACTH as well as β-endorphin [17]. Beta-endorphin acts as an endogenous analgesic during stress and is a natural component of parturition [18]. Jarvis et al. [18] demonstrated that sows penned in crates had higher levels of β-endorphin 48 h after farrowing compared to those in bedded pens. It is possible an acute stress event occurred in the present study, which may have caused an increased release of β-endorphin resulting in reduced oxytocin and more stillborn piglets. While stress metrics were not measured in the present study, it is likely that the sudden change of the light cycle in Dark sows resulted in an acute stress event. In a review by Castelhano-Carlos and Baumans [13], the authors state that changes of environmental lighting can cause distress in rats. In sheep, it has been shown that stress during parturition results in an increased chance of dystocia [19].

The present study reported that the duration of time spent standing, laying down, sitting, and kneeling were not affected by light treatment. Lachance et al. [20] similarly reported no differences in postural behaviors in sows with an extended photoperiod compared to sows exposed to 8 h of light per day during lactation. Likewise, Simitzis et al. [21] reported no postural differences in sows exposed to 8 h of light per day compared to those exposed to 20 h of light per day. The results of the present study are in agreement with these previous studies, indicating light treatment does not influence postural behaviors.

As mentioned previously, all sows were exposed to continuous light prior to this trial. The “free-running” circadian rhythm and sudden shift from that in the Dark treatment, may have altered the secretion patterns of melatonin. When comparing continuous light with the absence of light, Griffith and Minton [9] reported greater levels of melatonin in pigs exposed to constant light. Melatonin in pigs has also been shown to be important in early communication between the uterus and developing conceptuses [22]. In rats, photoperiod has been implicated as a regulator of the time of parturition [23,24]. Takayama et al. [24] suggested that the control of parturition time was through melatonin and its actions in the fetuses, although the authors stated the exact mechanism is unclear. Research has indicated that melatonin reduces the cortisol in dairy cows [25] and goats [26]. The change in light cycle for Dark sows in the present study may have altered the secretion pattern of melatonin and subsequently cortisol, which may partially explain the results observed. Unfortunately, samples were not taken to measure melatonin or cortisol in the present study, thus future studies are warranted to investigate the effect of shifting from continuous light to a 12 h light cycle on those hormone profiles.

## 5. Conclusions

Overall, light period available during farrowing only influenced the number of stillborn piglets and the time until the start of farrowing (length of gestation). Though light may be an influencer of farrowing behavior in sows, other stressful environmental factors should be considered. To further investigate the effects of isolation on farrowing, sound, pen size, human interaction, and lighting should all be considered in further planning.

## Figures and Tables

**Table 1:** Illuminance in farrowing crates with and without light deprivation tarps.

	Tarps Up		Tarps Down	
Height ^1^, cm	Light ^2^	Dark ^2^	Light ^2^	Dark ^2^
30.48	48.14 ± 5.41	31.93 ± 5.85	58.31 ± 3.86	0.12 ± 0.04
60.96	58.02 ± 14.45	45.99 ± 7.31	74.43 ± 9.89	0.13 ± 0.05
91.44	74.32 ± 8.05	51.87 ± 7.15	89.82 ± 8.55	0.22 ± 0.06

^1^ Illuminance was measured approximately 30.38 cm from the front of the crate in the center of the sow stall at three heights from the flooring (30.48, 60.96, and 91.44 cm). Illuminance was measured in 8 farrowing crates per treatment and data are presented as means ± standard deviation. ^2^ Light treatments included 24 h of light per day (Light) and 12 h light/dark per day (Dark). Light deprivation tarps were suspended from the ceiling and rolled up during the light phase of the light dark cycle and dropped down for the dark phase. While rolled up, the tarps hung approximately 30.5 cm from the ceiling, which resulted in some light being blocked by the tarps.

**Table 2:** Ethogram of behaviors observed during scan observations ^1^.

Postures	
Standing	All 4 legs are in contact with the ground and no portion of the abdomen is in contact with the floor
Lying	Ventral or lateral aspect of torso is in contact with the floor and no legs are actively weight bearing
Kneeling	Front body weight is supported by the carpal joints and hind legs remain in contact with the floor
Sitting	Front 2 legs are in contact with the ground while the hindquarters of the sow maintain contact with the floor
Posture Change	Count data; the number of times an animal transitioned from each posture to a new posture

^1^ Behaviors were observed in 5 min. scan intervals. Scans began two hours prior to farrowing and throughout the duration of farrowing for each sow.

**Table 3:** Effect of light treatment on farrowing performance.

	Treatments ^1^			
Measurement	Light	Dark	SE	*p*-Value
Gestation Length, d	117.2	116.4	0.8	0.026
Farrowing Start Time ^2^, h:min	13:22	13:40	1:48	0.906
Duration of Farrowing, min	301.9	247.6	52.7	0.393
Birthing interval between piglets, min	27.3	34.3	12.2	0.609
Total born, n	12.7	13.3	0.5	0.459
Liveborn, n	11.7	11.4	0.6	0.681
Stillborn, n	0.7	1.5	0.2	0.018
Mummified Piglets, n	0.3	0.4	0.2	0.932

^1^ Light treatments included 24 h of light per day (Light) and 12 h light/12 h dark per day (Dark) ^2^ Farrowing start time = average time of day that sows started giving birth as defined as the time at which the first piglet emerged from the sow.

**Table 4:** Effect of light treatment on sow behavior before and during farrowing ^1^.

	Treatment ^2^			
Behavior, Frequency	Light	Dark	SE	*p*-Value
Prior to farrowing ^3^				
Standing (%)	21.6	32.3	5.1	0.161
Laying Down (%)	70.9	62.0	5.6	0.274
Kneeling (%)	0.2	0.5	0.3	0.519
Sitting (%)	7.2	5.1	4.2	0.673
Posture Changes (#)	32.3	33.1	6.2	0.927
During farrowing				
Standing (%)	3.6	8.7	1.3	0.202
Laying Down (%)	93.6	88.5	3.2	0.279
Kneeling (%)	0.0	0.3	0.4	0.101
Sitting (%)	2.7	2.5	1.3	0.944
Posture Changes (#)	22.0	24.1	4.4	0.729

^1^ Video footage of sow farrowing evaluated using scan sampling therefore data are presented as percent of time spent performing a behavior. ^2^ Light treatments included 24 h of light per day (Light) and 12 h light/12 h dark per day (Dark). ^3^ Behavior observations performed for two hours prior to farrowing.

## Data Availability

Data are made available in the Illinois State University repository.
